# Influence of Chronic Exposure to Exercise on Heart Rate Variability in Children and Adolescents Affected by Obesity: A Systematic Review and Meta-Analysis

**DOI:** 10.3390/ijerph182111065

**Published:** 2021-10-21

**Authors:** Rodrigo M. Dias, Íbis A. P. Moraes, Maria T. A. P. Dantas, Deborah C. G. L. Fernani, Anne M. G. G. Fontes, Ana C. Silveira, Viviani Barnabé, Marcelo Fernandes, Patrícia M. Martinelli, Carlos B. M. Monteiro, David M. Garner, Luiz C. Abreu, Talita D. Silva

**Affiliations:** 1 Postgraduate Program in Medicine (Cardiology), Escola Paulista de Medicina, Federal University of São Paulo (EPM/UNIFESP), São Paulo 04021-001, Brazil; rmdias@unifesp.br (R.M.D.); ft.talitadias@gmail.com (T.D.S.); 2 Postgraduate Program in Rehabilitation Sciences, Faculty of Medicine, University of São Paulo (FMUSP), São Paulo 01246-903, Brazil; anne.m.gomes@hotmail.com (A.M.G.G.F.); carlosmonteiro@usp.br (C.B.M.M.); 3 Department of Health Sciences, University of the West of São Paulo (UNOESTE), Presidente Prudente 19050-680, Brazil; mariaterezaprado@hotmail.com (M.T.A.P.D.); deborah.fernani@gmail.com (D.C.G.L.F.); 4 Postgraduate Program in Physical Activity Sciences, School of Arts, Science and Humanities, University of São Paulo (EACHUSP), São Paulo 03828-000, Brazil; anaclara.silveira@hotmail.com (A.C.S.); marcelo.fernandes@mackenzie.br (M.F.); 5 Faculty of Medicine, City University of São Paulo (UNICID), São Paulo 03071-000, Brazil; vivianibarnabe@gmail.com; 6 Department of Physiotherapy, Mackenzie Presbyterian University, São Paulo 01302-907, Brazil; 7 Study Design and Scientific Writing Laboratory, ABC Medical School (FMABC), Santo André, São Paulo 09060-870, Brazil; martinelli_patricia@hotmail.com (P.M.M.); luiz.abreu@fmabc.br (L.C.A.); 8 Cardiorespiratory Research Group, Department of Biological and Medical Sciences, Faculty of Health and Life Sciences, Headington Campus, Oxford Brookes University, Gipsy Lane, Oxford OX3 0BP, UK; davidmgarner1@gmail.com

**Keywords:** exercise, obesity, autonomic nervous system, heart rate, physical fitness, disease prevention

## Abstract

Background: Sedentary lifestyles are increasingly common amongst children, and insufficient physical activity is a global epidemic estimated to contribute to future incapacities and potential deaths. Objective: We aimed to increase the amount of evidence concerning the effect of chronic exposure to exercise on heart rate variability in children and adolescents affected by obesity. Methods: A systematic review commenced following the PRISMA guidelines developed by Web of Science, Virtual Health Library, PubMed, Cochrane Library, Embase, Ovid, Medline Complete, and Scopus using keywords obtained from the Descriptors in Health Sciences and Medical Subject Headlines (MeSH) terms. We considered (1) Population: Pediatric individuals affected by obesity; (2) Intervention: Exercise; (3) Control: Pre-intervention and sedentary; (4) Outcomes: Clearly presented primary parameters; and (5) Studies: Clinical trials, case controls, case reports, and case series. Results: 11 articles were involved and predominantly included procedures observed during approximately 12 weeks with a distribution of three sessions per week, each session being 30–60 min of aerobic exercise; additionally, the exercise grades were typically completed at a percentage of subjects’ maximum heart rates. The meta-analyses displayed a significant effect on the domains of time (R-R interval, SDNN, rMSSD), frequency (HF ms^2^, HF (n.u.), LF/HF), and the non-linear index (SD1). Conclusions: Chronic exposure to exercise influences heart rate variability in children and adolescents affected by obesity by elevating the variability and parasympathetic activity and improving the sympathetic-vagal balance. Exercises should be recommended for the improvement of cardiac autonomic modulation to prevent the likelihood of further chronic diseases.

## 1. Introduction

Obesity is problematic and affects a large section of the global population [1]. The World Health Organization (W.H.O.) states that amongst children and adolescents, obesity has increased tenfold in the last four decades, and by 2022, there will be more children and adolescents affected by obesity than presenting with malnutrition [2]. Childhood obesity has been linked to the early onset of various chronic conditions, such as type 2 diabetes and systemic arterial hypertension [3]. Additionally, research demonstrates that obesity is often attended by earlier dysfunction of the autonomic nervous system [4]. Adverse obesity-related health consequences have been associated with possible cognitive decline and increased body mass index, while central adiposity has been shown to be correlated with performance impairments under task conditions that require executive control [5]. Young individuals affected by obesity suffer from a decline in cardiopulmonary function, poor exercise tolerance, and low self-esteem [6].

Although treatment choices include medication, diet changes, intensive behavioral modification, and behavioral therapy, the use of exercise provides an interestingly different impact [7,8]. Sedentary lifestyles are increasing amongst children, and insufficient physical activity is a global epidemic estimated to contribute to future incapacities and potential deaths [9,10].

Gonzáles–Ruiz’s [11] systematic review of the use of exercise to reduce fat in those with pediatric obesity supports the current approval for physical, mainly aerobic exercise as an effective intervention against non-alcoholic fatty liver disease progression by targeting hepatic lipid composition and visceral and subcutaneous adipose tissue. Likewise, the impact of exercise causes enhancements in autonomic modulation [12,13,14], and this can affect obesity [15,16]. Although there is an existing association between autonomic modulation, exercise, and obesity, the application of exercise as a treatment method for probable cardiac autonomic dysfunction generated by obesity in pediatric individuals necessitates further study [17].

One of the most effective ways to judge these autonomic dysfunctions is via the study of heart rate variability (HRV), an important predictor of cardiovascular health [18,19] above being a simple, reliable, and non-invasive way of scrutinizing autonomic function [20]. Similarly, cardiac autonomic dysfunction, as indicated by a reduced HRV, can promote inferior cardiovascular fitness outcomes and diminished parasympathetic function [3,21,22].

A systematic review of cross-sectional studies was performed by Oliveira et al. [23] in an attempt to determine whether cardiac autonomic function is linked to cardiorespiratory fitness and physical activity in children and adolescents. They demonstrated that physical activity was positively associated with parasympathetic activity but that most studies had a small amount of evidence. Consequently, there is a need for studies that elevate the level of knowledge concerning the effect of chronic exposure to exercise on the HRV of pediatric individuals affected by obesity. According to Farrell and Turgeon [24], after 30 days of exposure to exercise, there are chronic physiological adaptations. Based on the above-mentioned information, the study objective here was to evaluate the influence of chronic exposure to exercise on the HRV of children and adolescents affected by obesity and to specify exercise as an enhancer for combating potential autonomic dysfunctions caused by obesity.

## 2. Materials and Methods

The review was conducted according to the guidelines of the Preferred Reporting Items for Systematic Reviews and Meta-Analyses (PRISMA) [25,26,27,28,29,30,31]. The protocol for this review was registered in PROSPERO under registration number CRD42020178328.

### 2.1. Research Strategy

For this systematic review, studies were investigated in the databases Web of Science (WOS), Virtual Health Library (*Biblioteca Virtual em Saúde*-BVS), PubMed, Cochrane Library, Embase (Elsevier), Ovid, Medline Complete (EBSCO), and Scopus (Elsevier) during January 2020 using keywords obtained from the Descriptors in Health Sciences (DeCS) of the Virtual Health Library and MeSH. These database searches were organized according to the PICOS model (Population, Intervention, Control, Outcome, Study design) [32]. The search was performed using the terms “Heart Rate Variability AND Exercise AND Obesity AND (Child OR Children OR Adolescent OR Adolescents)” and “Heart Rate Variability AND Exercise AND (Pediatric Obesity OR Childhood Obesity)” (Table S1—Supplementary Materials).

### 2.2. Inclusion and Exclusion Criteria for Selection of Studies

These articles were selected according to the following inclusion criteria: (1) Population: Pediatric individuals affected by obesity; (2) Intervention: Exercise; (3) Control: Pre-Intervention; (4) Outcomes: Evidently presented primary parameters; and (5) Studies: Clinical trials, case controls, case reports, and case series. The obesity criteria (relative to normative data for age, gender, and ethnicity) were represented by the ≥85th percentile of the updated growth norm of each different country and/or the z-BMI score from 2 to 3 [33,34]. There were no restrictions of dates in the inclusion of publications.

Accordingly, the following items were omitted: (1) abstracts and expanded abstracts; (2) complete articles that were not written in English, Portuguese, or Spanish; and (3) articles that did not refer to both exercise and pediatric obesity. In cases of discrepancy, a third author was consulted. Duplicate records and studies that were unrelated to the planned objectives were excluded.

### 2.3. Data Extraction

The data were extracted from the included studies via an electronic spreadsheet. Data were collected on the study design, sample number, intervention time, type of exercise, and device used to collect HRV, all following the PICOS structure. Additionally, numerical data for further meta-analyses were extracted to consider the means and standard deviations presented by the studies. To extract data from studies that presented results in the form of graphs, WebPlotDigitizer [35] was used to find the central and dispersion measures.

### 2.4. Parameters of Interest

The parameters of interest originated from the three possibilities of comparison and outcome: (1) Post-Intervention vs. Pre-Intervention; (2) Obese with exercise vs. Obese without exercise; and (3) Obese with exercise vs. Not obese with exercise. Next were the variables related to HR and HRV in the time domains: (1) RR interval: interval between consecutive heartbeats; (2) SDNN: standard deviation of all normal RR intervals recorded in a time interval, expressed in ms; and (3) rMSSD: the root-mean-square of differences between adjacent normal RR intervals in a time interval, expressed in ms. Additionally, we defined frequency domains: (4) HF: High Frequency, extending from 0.15 Hz to 0.40 Hz, expressed in milliseconds squared and normalized units; (5) LF/HF ratio: reflects the sympathetic-vagal balance]; and the non-linear metrics; (6) SD1: represents the dispersion of points perpendicular to the line of identity, short term variability of continuous RR intervals; and (7) SD2: long-term variability of continuous RR intervals. According to Vanderlei et al. [18], these are the main parameters analyzed, and for that reason, these parameters of interest were chosen. The secondary parameters were body mass index (kg/m^2^) (BMI) and body fat percentage (Fat%).

### 2.5. Evaluation of Risk of Bias

The PEDro scale [36,37] was obligatory in evaluating the quality of the evidence of the studies, as it is the most often used scale in the rehabilitation area. This scale was developed by the Physiotherapy Evidence Database to evaluate experimental studies that can attain an overall score of 10 points. These criteria are confined in the Delphi list and are useful in investigating items in systematic reviews consistent with their proposed methodology, considering the studies as presenting the following amounts of evidence: “excellent” being 9–10, “good” being 6–8, “reasonable” being 4–5, and “poor” being less than 4.

### 2.6. Statistical Analyses

Review Manager Software 5.3 was essential in completing the meta-analysis calculations [38]. The data used were those expressed as the mean and standard deviation. If such data were presented as median and interquartile ranges, their means and standard deviations were estimated according to the method by McGrath et al. [39]. Additionally, dispersion measures of standard error were transformed into the standard deviation.

To study the inverse variances, the means and standard deviations of the results of each study were required. The fixed effects of the treatments were studied; hitherto, in the case of significant heterogeneity between studies, random effects were analyzed. Heterogeneity was considered using the Q2 and I2 tests. Studies that contained an excess of one group that used exercise as a treatment were combined into one group using the algorithm described by Cochrane [40].

## 3. Results

**Figure 1:**
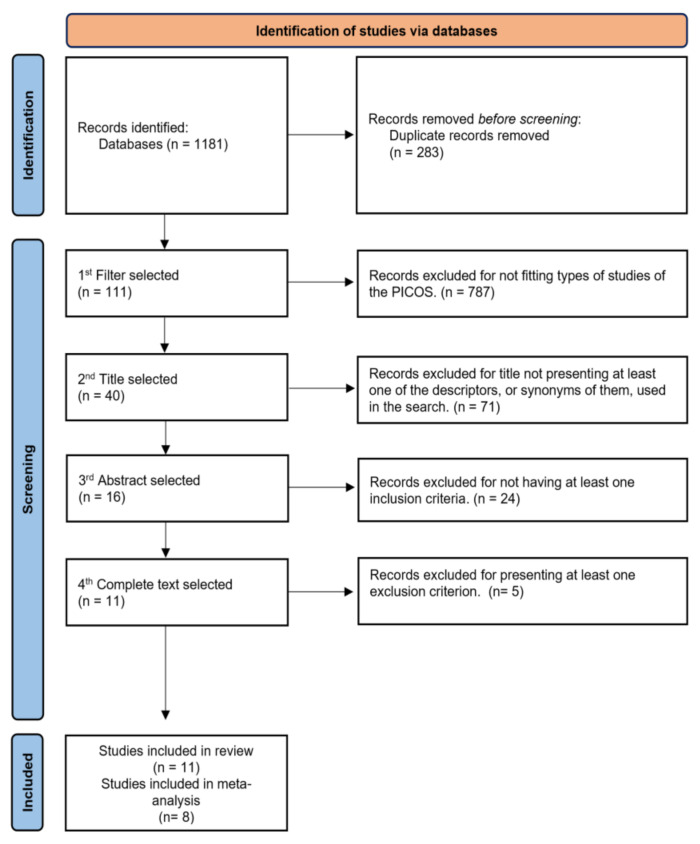
Flowchart.

The literature research (5–30 January 2020) consisted of a total of 1181 potentially relevant articles, of which 283 were duplicates. That left 787 viable studies, of which 111 turned out to be relevant studies. After scrutinizing the titles, 71 studies were excluded. Twenty-four more studies were excluded after investigating the abstracts, and 12 additional studies were eliminated after analyzing the full texts. In the succeeding step, the three filters were applied, resulting in 11 articles being included in this review. The studies’ flowchart and inclusion strategy are exemplified in Figure 1. Amongst the 11 studies [3,5,6,17,21,41,42,43,44,45], a total of 398 individuals were included as a sample, comprising 176 boys and 222 girls aged 5 to 18 years, as illustrated in Table 1.

### 3.1. Study Methods

The study methods are demonstrated in Table 1. Amongst the procedures, the ensuing factors were predominant: lasting approximately 12 weeks, distributed between three sessions per week, and 30 to 60 min per session. The principal type of exercise enforced was aerobic exercise, and the exercise grade was usually performed at a percentage (%) of the maximum HR. HRV collection was usually completed before and after the intervention by means of a Polar^®^ heart rate monitor.

### 3.2. Main Study Outcomes

With regard to the main outcomes achieved in the studies, significant changes in HRV were observed after exercise intervention. This factor can be perceived after evaluating the results of the HR meta-analysis and HRV indices in the time domain, frequency domain, and non-linear metrics.

### 3.3. Risk of Bias

The classification of studies exhibited a majority to be reasonable to poor, as illustrated in Table 1.

### 3.4. Meta-Analyses

It was plausible to perform only meta-analyses of the HRV index “Post-Intervention vs. Pre-Intervention” (1) because of the ways of presenting the data of the selected articles. Yet since it is imperative to highlight in this paper, when possible, the variables also related to the other two possibilities of comparison and outcomes discussed, “Obese with exercise vs. Obese without exercise” (2) and “Obese with exercise vs. Not obese with exercise” (3).

**Figure 2:**
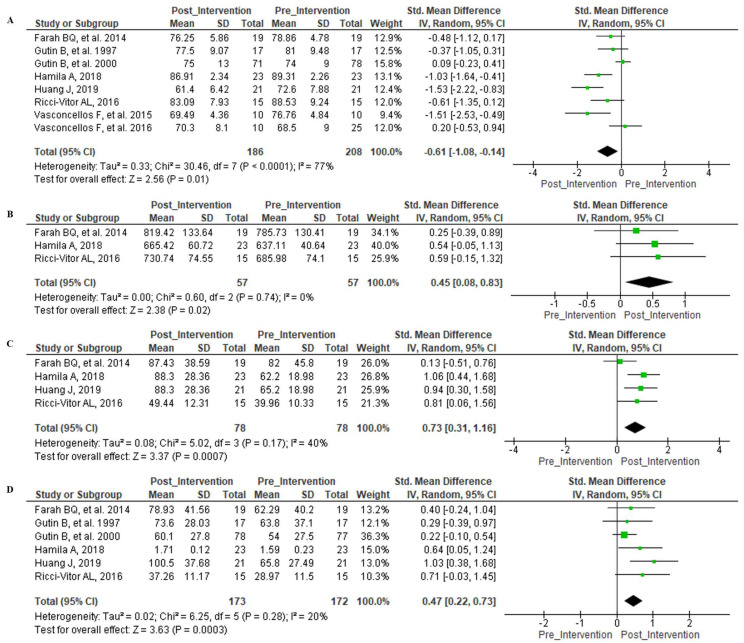
Forest Plot—Time domain indices. Legend—(**A**): Mean Heart Rate mean; (**B**): R-R interval; (**C**): SDNN; (**D**): rMSSD.

Figure 2A determines that exercise had an important effect on the HR index of individuals both post-intervention and pre-intervention, but this presented significant heterogeneity of data.

Figure 2B establishes that the mean RR interval caused a significant effect (Z = 2.38 (*p* = 0.02)) without significant heterogeneity. For the HRV indices related to the time domain, the SDNN index in Figure 2C displayed a significant effect when comparing post-intervention and pre-intervention (Z = 3.37 (*p* = 0.0007)) without significant heterogeneity. In Figure 2D, the HRV rMSSD index was considered for both post-intervention and pre-intervention and established a significant effect (Z = 3.63 (*p* = 0.0003)) while not showing significant heterogeneity.

**Figure 3:**
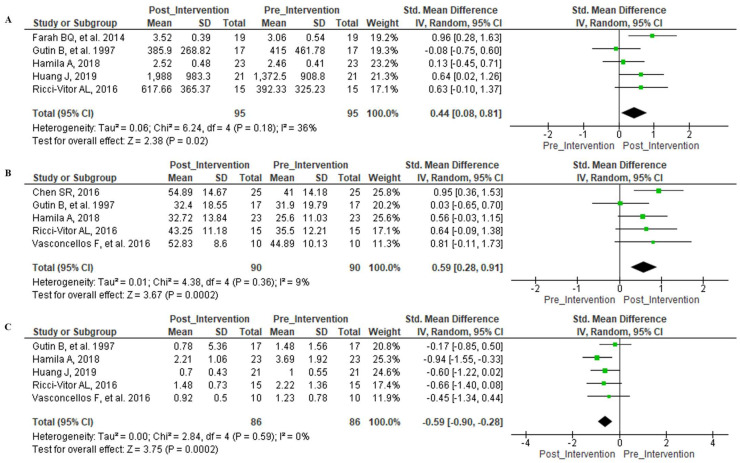
Forest Plot—Frequency Domain indices. Legend—(**A**): HF ms^2^; (**B**): HF n.u.; (**C**): LF/HF ratio.

For the HRV indices related to the frequency domain, the HF index (ms) presented a significant effect post-intervention as opposed to pre-intervention (Z = 2.38 (*p* = 0.02)) and also lacked significant heterogeneity (Figure 3A). The HF (n.u.) index and post-intervention vs. pre-intervention both established an important effect (Z = 3.75 (*p* = 0.0002)) but did not display significant heterogeneity (Figure 3B). When investigating the LF/HF ratios, in both post-intervention and pre-intervention there was an important effect (Z = 3.75 (*p* = 0.0002)) with an absence of significant heterogeneity (Figure 3C).

**Figure 4:**
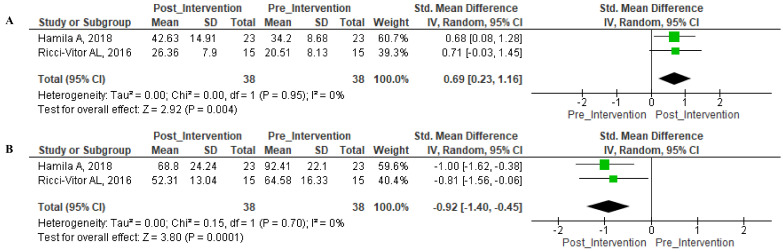
Forest Plot—Non-linear indices. Legend—(**A**): SD1; (**B**): SD2.

The non-linear metric SD1 was equated for both post-intervention and pre-intervention, with both indicating a significant effect (Z = 2.92 (*p* = 0.004)) without significant heterogeneity (Figure 4A). SD2 index had a negative effect (Z = 3.80 (*p* = 0.0001)) for both post-intervention and pre-intervention without significant heterogeneity (Figure 4B).

**Figure 5:**
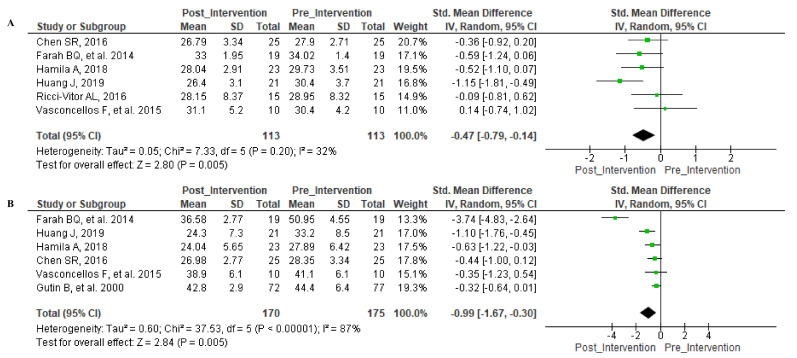
Forest Plot—Secondary parameters. Legend—(**A**): BMI; (**B**): Fat%.

The secondary parameters were evaluated, and the forest plot was illustrated in Figure 5. In Figure 5A, the exercise offered a significant effect (Z = 2.80 (*p* = 0.005)) in the reduction in BMI (kg/m^2^) without significant heterogeneity. In the same way, Fat% demonstrated a significant change (Figure 5B) (Z = 2.84 (*p* = 0.005)) but not a significant heterogeneity (I^2^ = 87%; *p* < 0.00001)).

## 4. Discussion

The study objective was to identify the effect of chronic exposure to exercise on HRV in children and adolescents affected by obesity. Even though the classification of studies exhibited a majority to be reasonable to poor, the same tendencies in the results were found, in agreement with previous findings regarding the time domains of HRV. We revealed that children and adolescents affected by obesity in the intervention group have a reduced mean HR post-exercise compared with pre-intervention and that this is related to a greater mean RR interval. Moreover, children and adolescents affected by obesity in the intervention group established greater variability post-exercise within the time-series of heartbeats, as revealed by the SDNN index, and higher parasympathetic activity, as expressed by rMSSD.

Similarly, in the frequency domain, when examining the HF ms^2^ in (n.u.), a pattern of increase was revealed in post-exercise intervention as well as a reduction in the LF/HF index post-exercise intervention when equated to pre-intervention, indicating increased parasympathetic activity and improved sympathetic-vagal balance, respectively. Still, no significant results were observed while considering the LF ms^2^, even if in normalized units, a reduction was identified post-exercise intervention. It is recognized that LF reflects both sympathetic and vagal influence and has been consistent with baroreflex sensitivity [47]. These results were undetected in LF (n.u.) and HF (n.u.) when related to pre-intervention.

Finally, in the chaotic (or non-linear) domain, SD1 showed a pattern of increased post-exercise intervention in the exercise group when compared with pre-intervention, therefore representing an increase in parasympathetic activity since, physiologically, the transverse axis (SD1) is a measurement of short-term changes in the RR intervals, which are considered an indicator of parasympathetic activity. A contradictory pattern (decrease) was revealed in SD2. The physiological rationale of the longitudinal axis (SD2) is not as noticeable, yet it is thought that it reflects the long-term changes in RR intervals, and these assumptions are open to different explanations, such as sympathetic input with parasympathetic influences [48].

Even supposing that the effects of chronic exposure to exercise on sympathetic activity remains questionable, the exercise demonstrably promoted greater parasympathetic cardiac activation. Perhaps this is the crucial indicator of positive exercise adaptation [5], demonstrated by the rMSSD, HF, and SD1 indices. In this manner, chronic exposure to exercise could impart improved cardiovascular physiological health [3,41], thereby reducing autonomic dysfunction [17], improving cardiac electrical stability, protecting against experimentally induced myocardial infarction [42], and elevating arterial baroreflex sensitivity and cardiorespiratory functional capacity [21].

However, intervention programs proposing activities with lower intensity did not have such advantageous effects. This is an important issue, as exercise intensity appears to be a determinant in HRV response [3,6,22,43]. The physical activity programs of the studies included in this review enforced many types of exercise. Other than aerobic training [3,5,6,21,41,42,43,44], the studies involved sports, commonly including soccer practice [45,46], and a solitary research study that assessed resistance training, conducted by Farinatti et al. [17]. Notably, it was revealed that the training time—not only the session time but also the duration time in weeks—is foremost in maintaining the useful effect of exercise, as once the training periods ceased, the indices tended to slowly return to previous values [42].

Other confounders, e.g., dietary control, appear to have not influenced the evaluated studies; in most studies, while no lifestyle or any form of nutritional counseling was provided, positive results were attained. Two studies proposed dietary counseling, yet there was no attempt to reduce energy intake [3,6]; additionally, two studies associated exercise intervention with a dietary restriction [21,44]. If obesity produces an imbalance between dietary intake and energy expenditure, it appears that the latter found positive changes that were enhanced by calorific restriction. Despite this, a lessening in BMI was achieved in almost all studies, and a decrease in fat percentage in all, so we can conclude that the physical activity program completed in the studies cut the subjects’ obesity status. Hence, this may also be a feature that influenced changes in HRV.

It is important to remark that the autonomic nervous system directs voluntary and involuntary physiological processes, such as digestion, blood pressure, hormonal regulation, energy metabolism, and heart rate, and is accordingly considered an important regulator of homeostasis. It innervates fat depots, which are associated with catecholamine production. Thus, the biological mechanisms could involve adipocytokines secreted by fat cells [49]. According to these statements, studies have revealed that body composition measures are negatively associated with HRV parameters and indicators of parasympathetic activity [50]. RMSSD (parasympathetic activity) was negatively correlated with fat mass [51] and, together with weight loss [52], was linked to a decrease in HR and an increase in HRV (as indicated by SDNN). Other than that, a calorific restriction has been confirmed as an intervention that may reverse the autonomic changes [53].

In the study of Veijalainen et al. [54], physical activity was related to better cardiac autonomic nervous system function in children independent of gender, adiposity, and the clustering of cardiometabolic risk factors. The authors comment that hemodynamical regulation of the human body is complex and involves neural and humoral mechanisms; hence, one of the explanations for the observed associations of lower physical activity with lower HRV could be that it decreases blood volume and left ventricular stroke volume to result in an increased heart rate on account of increased sympathetic activity.

Aerobic exercise training has been stated as an integral component of interventions to reduce obesity and related co-morbidities in children and adolescents [3,6]. In addition, physical activity and dietary interventions are beneficial for glucose metabolism, skeletal muscle function, bone stability, psychological well-being, and physiological organ functions [21]. In contrast, there is evidence that lifestyle interventions that improve weight status and metabolic risk may improve autonomic dysfunction in children affected by obesity [17,44]. Despite the current evidence connecting exercise training to enhanced autonomic nervous system activity in children, the exact amount of exercise required for optimal adaptation is indeterminate.

As HRV declines with age [42], it is easy to speculate that the promotion of physical activity should be recommended, as it can improve autonomic cardiac modulation. Such activity would aid in preventing chronic diseases, such as diabetes, cardiac events, stroke, and so forth, to accordingly lead to a better quality of life and extended life expectancy over time. Above and beyond these, this practice would affect reductions in public health expenditure through the reduction in the prevalence of chronic diseases.

From a methodological perspective, we did not address the impact of the method of HRV capture, but we can affirm that most studies used the Polar heart rate monitor to capture RR intervals [3,6,8,17,21,40,41,42] and the Kubios HRV^®^ software to compute the indices [3,6,44,45,46]. Additionally, the times and sample rates of recording were not standardized in the studies. This is a key fact, considering that data standardization in one study included in the meta-analysis used a different method of data conversion; in Hamila et al. [38], RMSSD, LF (n.u.), and HF (n.u.) were logarithmically transformed to normalize data. Another important point to be revealed is that the lack of knowledge about the HRV indices can generate errors in the interpretation of the data, as realized in the study of Chen et al. [5], in that the authors state that there was an increase in HF (n.u.) and LF (n.u.) after exercise. However, since these indices represent the quantity of each one (LF or HF) concerning the whole (total power), as one index increases, the other must inevitably decrease.

One of the major restrictions of the current analysis is the fact that several studies had to be excluded as a result of insufficient reporting of descriptive data. Accordingly, we encourage researchers to provide descriptive statistics for HRV to further augment the existing body of scientific research. Additionally, the sample sizes of the studies were small, and there was heterogeneity in the study samples, the numbers of participants, groups, and grades of exercise intensity, making it problematic to extrapolate the data. Finally, the non-differentiation between ethnicities could be an influencing factor on the data evaluated in the studies. Therefore, more robust studies are required to establish whether these effects can be replicated.

## 5. Conclusions

We conclude that chronic exposure to exercise appears to influence HRV in children and adolescents affected by obesity by increasing variability (SDNN index) and parasympathetic activity (expressed by rMSSD, HF, and SD1), cultivating the sympathetic-vagal balance demonstrated by a decrease in LF/HF. Nonetheless, the evidence is reasonable to poor and should be reassessed with more robust clinical studies in future research. Still, exercise can be recommended for the improvement of cardiac autonomic modulation to decrease the likelihood of further appearances of chronic diseases.

## Figures and Tables

**Table 1:** Characteristics of the studies.

**Characteristics of the Samples**						
**Author**	**N**	**Age (Years)**	**Sex (Males)**	**BMI (kg/m^2^)**	**SD**	**PEDro Score**
Chen S. R. et al., 2016 [5]	50	±12	28	Control: 27.9 ± 2.71	RCT	05/10
				Experimental: 26.79 ± 3.34		
Farah B. Q. et al., 2014 [3]	43	13–18	14	Controls: HIT = 33.5 ± 1.4; LIT = 34.5 ± 1.3	RCT	05/10
				Experimental: HIT = 32 ± 1.8; LIT = 33.9 ± 1.7		
Farinatti P. et al., 2016 [17]	44	13–17	20	Control: 20.6 ± 2.4	CT	04/10
				Experimental: 32.1 ± 3.6		
Gutin B. et al., 1997 [41]	35	7–11	12	Control: 28.8 ± 1.6	RCT	05/10
				Experimental: 31.4 ± 1.8		
Gutin B. et al., 2000 [42]	79	±10	26	-	RCT	03/10
Hamila A. et al., 2018 [6]	31	±14	12	-	RCT	04/10
Huang J. et al., 2019 [21]	21	5–16	11	Control: 30.4 ± 3.7	CT	04/10
				Experimental: 26.4 ± 3.1		
Paschoal M. A. et al., 2018 [43]	15	9–12	-	Baseline: 23.20 ± 1.3	CT	03/10
Ricci-Vitor A. L. et al., 2016 [44]	15	10.93 ± 2.28	7	Control: 28.95 ± 8.32	CT	04/10
				Experimental: 28.15 ± 8.37		
Vasconcellos F. et al., 2015 [46]	35	±14	26	-	RCT	05/10
Vasconcellos F. et al., 2016 [45]	30	12–2017	20	Control: 32.2 ± 4.9	RCT	05/10
				Experimental: 31.1 ± 5.2		
**Methods of Included Studies**						
**Author**	**Intervention Time**	**Exercise Type**	**Control Group**	**Graduation Method of Exercise Intensity**	**Main Instrument of HRV**	
Chen S. R. et al., 2016 [5]	12 wk	Aerobic	No exercise	% HR max	CheckMyHeart 3.0, DailyCareBioMedical, Inc., Taoyuan, Taiwan	
Farah B. Q. et al., 2014 [3]	24 wk	Aerobic	-	Metabolic equivalent of task (MET) and VO_2_Max	Polar model RS800CX, Polar Electro Inc., Lake Success, New York, NY, USA	
Farinatti P. et al., 2016 [17]	30–40 min/3 d/12 wk	Resistance training	Normal weight	% Maximum Repetition	Finometer, Finapres Medical Systems, Amsterdam, The Netherlands	
Gutin B. et al., 1997 [41]	40 min/5 d/16 wk	Aerobic	No exercise	% HR Submax	Polar Vantage, Port Washington, New York, NY, USA	
Gutin B. et al., 2000 [42]	40 min/5 d/16 wk	Aerobic	-	HR	Polar Vantage, Port Washington, New York, NY, USA	
Hamila A. et al., 2018 [8]	60 min/3 d/8 wk	Aerobic	No exercise	Maximal aerobic speed	Polar RS810, Kempele, Finland	
Huang J. et al., 2019 [21]	5 h/6 d/6 wk	Aerobic	-	% HR max	Sphygmo Corsystem, AtCor Medical, Sydney, Australia	
Paschoal M. A. et al., 2018 [40]	12 sessions/40 min	Aerobic	-	% HR Submax	Polar S810^®^, Kempele, Finland	
Ricci-Vitor A. L. et al., 2016 [44]	60 min/3 d/12 wk	Aerobic	-	Not graduated	Polar Electro, model S810i, Kempele, Finland	
Vasconcellos F. et al., 2015 [46]	60 min/3 d/12 wk	Sport	No exercise/Normal weight	HRV	Polar RS800cx, Polar^TM^, Kempele, Finland	
Vasconcellos F. et al., 2016 [45]	60 min/3 d/12 wk	Sport	No exercise/Normal weight	Not graduated	Polar RS800cx, Polar^TM^, Kempele, Finland	

Legend—SD: Study Design; RCT: randomized controlled trial; CT: Clinical Trial; wk: weeks; min: minutes; d: days; BMI: Body Mass Index; HIT: High-Intensity Interval Training; LIT: Low-Impact Interval Training.

## Supplementary Materials

The following are available online at https://www.mdpi.com/article/10.3390/ijerph182111065/s1, Table S1: Search Strategy.
