# Supplementary material for: Influence of Chronic Exposure to Exercise on Heart Rate Variability in Children and Adolescents Affected by Obesity: A Systematic Review and Meta-Analysis
Source: Int J Environ Res Public Health. 2021 Oct 21;18(21):11065. doi: 10.3390/ijerph182111065 (PMC8583488; doi:10.3390/ijerph182111065)
Supplement: Supplementary file 1 [file ijerph-18-11065-s001.zip › ijerph-1340695-supplementary.pdf]

Table S1. Search Strategy.

Mesh Terms	*Database*	*Combination*	*Search strategy*
Heart Rate Variability	Embase (Elsevier)	Heart Rate Variability AND Exercise AND Obesity AND (Child OR Children OR Adolescent OR Adolescents); Heart Rate Variability AND Exercise AND (Pediatric Obesity OR Childhood Obesity)	Search strategy 1: ('heart rate variability'/exp OR 'heart rate variability' OR (('heart'/exp OR heart) AND rate AND ('variability'/exp OR variability))) AND ('exercise'/exp OR exercise) AND ('obesity'/exp OR obesity) AND ('child'/exp OR child OR 'children'/exp OR children OR 'adolescent'/exp OR adolescent OR 'adolescents'/exp OR adolescents);<br>Search strategy 2: heart AND rate AND variability AND exercise AND (pediatric AND obesity OR childhood) AND obesity
	Ovid		Search strategy 1- (Heart Rate Variability and Exercise and Obesity and (Child or Children or Adolescent or Adolescents)).mp. [mp=title, abstract, full text, caption text]<br>Search strategy 2- (Heart Rate Variability and Exercise and (Pediatric Obesity or Childhood Obesity)).mp. [mp=title, abstract, full text, caption text]
	Medline Complete (EBSCO)		Search strategy 1- Heart Rate Variability AND Exercise AND Obesity AND (Child OR Children OR Adolescent OR Adolescents)<br>Search strategy 2- Heart Rate Variability AND Exercise AND (Pediatric Obesity OR Childhood Obesity)
	Scopus (Elsevier)		Search strategy 1- TITLE-ABS-KEY (heart AND rate AND variability AND exercise AND obesity AND (child OR children OR adolescent OR adolescents))<br>Search strategy 2- TITLE-ABS-KEY (heart AND rate AND variability AND exercise AND (pediatric AND obesity OR childhood AND obesity))
	PubMed		Search strategy 1: (("heart rate"[MeSH Terms] OR ("heart"[All Fields] AND "rate"[All Fields]) OR "heart rate"[All Fields]) AND Variability[All Fields]) AND ("exercise"[MeSH Terms] OR "exercise"[All Fields]) AND ("obesity"[MeSH Terms] OR "obesity"[All Fields]) AND (("child"[MeSH Terms] OR "child"[All Fields]) OR ("child"[MeSH Terms] OR "child"[All Fields]

; Exercise; Obesity; Child; Children; Adolescent; Adolescents; Pediatric Obesity; Childhood Obesity.			OR "children"[All Fields]) OR ("adolescent"[MeSH Terms] OR "adolescent"[All Fields]) OR ("adolescent"[MeSH Terms] OR "adolescent"[All Fields] OR "adolescents"[All Fields]))<br>Search strategy 2: (("heart rate"[MeSH Terms] OR ("heart"[All Fields] AND "rate"[All Fields]) OR "heart rate"[All Fields]) AND Variability[All Fields]) AND ("exercise"[MeSH Terms] OR "exercise"[All Fields]) AND (("pediatric obesity"[MeSH Terms] OR ("pediatric"[All Fields] AND "obesity"[All Fields]) OR "pediatric obesity"[All Fields]) OR ("pediatric obesity"[MeSH Terms] OR ("pediatric"[All Fields] AND "obesity"[All Fields]) OR "pediatric obesity"[All Fields] OR ("childhood"[All Fields] AND "obesity"[All Fields]) OR "childhood obesity"[All Fields])).
	BVS		Search strategy 1: Heart Rate Variability AND Exercise AND Obesity AND (Child OR Children OR Adolescent OR Adolescents);<br>Search strategy 2: heart rate variability AND exercise AND (pediatric obesity OR childhood obesity)
	Cochrane Library		Search strategy 1: Heart Rate Variability AND Exercise AND Obesity AND (Child OR Children OR Adolescent OR Adolescents) in Title Abstract Keyword - (Word variations have been searched);<br>Search strategy 2: Heart Rate Variability AND Exercise AND (Pediatric Obesity OR Childhood Obesity) in Title Abstract Keyword - (Word variations have been searched).
	WOS		Search strategy 1: (Heart Rate Variability AND Exercise AND Obesity AND (Child OR Children OR Adolescent OR Adolescents)).<br>Allotted time: Every year. Indices: SCI-EXPANDED, SSCI, A&HCI, CPCI-S, CPCI-SSH, ESCI;<br>Search strategy 2:(Heart Rate Variability AND Exercise AND (Pediatric Obesity OR Childhood Obesity)).<br>Allotted time: Every year. Indices: SCI-EXPANDED, SSCI, A&HCI, CPCI-S, CPCI-SSH, ESCI.

Legend: BVS: Virtual Health Library; WOS: Web of Science; MeSH: Medical Subject Headings; SCI-EXPANDED: Science Citation Index Expanded; SSCI: Social Sciences Citation Index; A&HCI: Arts & Humanities Citation Index; CPCI-S: Conference Proceedings Citation Index - Science; CPCI-SSH: Conference Proceedings Citation Index - Social Sciences & Humanities; ESCI: Emerging Sources Citation Index.
